# Life is a journey

**DOI:** 10.1186/s40634-019-0202-8

**Published:** 2019-07-11

**Authors:** Henning Madry

**Affiliations:** Lehrstuhl für Experimentelle Orthopädie und Arthroseforschung, Saarland University, Kirrberger Strasse, Building 37, 66421 Homburg/Saar, Germany

*Siempre imaginé que el Paraíso sería algún tipo de biblioteca.I have always imagined that Paradise will be a kind of library.*Jorge Luis Borges (1899 - 1986)

The Journal of Experimental Orthopaedics was launched in late 2013 by the European Society of Sports Traumatology, Knee Surgery & Arthroscopy (ESSKA), serving to bridge the gap between orthopaedic basic science and clinical relevance. I was provided with the good fortune to envision and serve as the founding Editor-in-Chief of the Journal of Experimental Orthopaedics. Leading the Journal has been an extremely rewarding and memorable phase of my professional career.

From early on, the vision of a new Journal was supported and mentored by creative leaders of the ESSKA, among which Lars Engebretsen, João Espregueira-Mendes, Romain Seil, Niek van Dijk, Matteo Denti, David Dejour, Jacques Menetrey and many others. The idea took on a real form on March 5th, 2011 in Salon 3 of the Villa Sarasin [home of the late Emile Édouard Sarasin (1843–1917), a swiss scientist] in Le Grand-Saconnex near Geneva in Switzerland at an ESSKA Board Meeting. There, it was decided to proceed with the launch of a new journal that would be the official basic science journal of ESSKA, complementing the more clinical Knee Surgery, Sports Traumatology, Arthroscopy (KSSTA) journal. The Journal of Experimental Orthopaedics was designed as a unique biomedical journal with a surgical background, aiming to merge the rigor of basic science with the art of orthopaedic surgery (Madry, 2014).

Our 100th article appeared in Pubmed on 4 September 2017, and at the time of this Editorial, nearly 190 articles have been published. Successfully attracting good quality manuscripts, often with the help of active ESSKA committees, significantly promoted the journal’s reputation. The Journal of Experimental Orthopaedics guarantees a rigorous, objective, and timely peer review process. Editorial decisions are solely based on scientific integrity and merit, not profit or scientific fashion. Although an evolution of the journal is inevitable, these fundamental principles shall never change for the Journal of Experimental Orthopaedics to retain its stature. In a recent Clarivate analysis, the journal would be placed in the second quartile (Q2) and therefore in the upper half of journals for the category “Orthopaedics - Sci” category in Web of Science. The open access model allowed for a remarkable dissemination: for example a study on determining bone tunnel sizes in anterior cruciate ligament reconstruction has been accessed about 34.500 times since its publication in June 2014 (Crespo et al., 2014). Moreover, the already classical review on the role of aggrecan in normal and osteoarthritic cartilage, written by the late Peter J. Roughley (1947–2018), is with 45 citations since its publication in July 2014 our most cited article ever (Roughley & Mort, 2014). Although the journey began nearly exactly 5 years ago, it seems like only yesterday when our first article was published on 26 June 2014 (Brinkman et al., 2014).

The time has now come to hand over the responsibility at the helm, as Ralph Waldo Emerson (1803–1882) famously quoted: “Life is a journey, not a destination.” It was not an easy decision for me to make a move to a newly introduced journal, and although I will be taking up a new assignment as Editor-in-Chief of Osteoarthritic and Cartilage Open, I will continue to support the Journal of Experimental Orthopaedics to the best of my ability. Please join me in welcoming Stefano Zaffagnini in his new capacity as Editor, and I am sure that Stefano with his superb clinical and scientific expertise brings excellent credentials that will continue to provide the dedication, enthusiasm, and creativity for the continued stewardship of the journal.

At the same time, I would like to express my sincere appreciation to the ESSKA leadership who I thank for giving me the editorial responsibility to establish the Journal of Experimental Orthopaedics. I am very grateful for the support of our excellent Editorial Board and distinguished Board of Trustees, among which scientists serving as presidents of universities or international organizations such as ESSKA, the ORS, and the ICRS, and to other Editors-in-Chief, among which Jón Karlsson and Ejnar Eriksson of KSSTA, Bruce Reider of The American Journal of Sports Medicine, Farshid Guilak of the Journal of Biomechanics, Detlev Ganten of the Journal of Molecular Medicine, and Rocky Tuan of Stem Cell Research & Therapy. I have highly enjoyed working very effectively with our Editorial Office in Luxembourg served by a tireless staff of dedicated professionals, and Gabriele Schröder in her position as Editorial Director at SpringerNature, and I would like to thank them for their constant effort. Finally yet importantly, I thank our authors, reviewers, and readers for ensuring the success of the Journal of Experimental Orthopaedics.

I wish you all the best.

Sincerely,

Henning Madry
